# Supplementary material for: The transfer of antibiotic resistance genes between evolutionarily distant bacteria
Source: mSphere. 2025 Jun 3;10(6):e00114-25. doi: 10.1128/msphere.00114-25 (PMC12188727; doi:10.1128/msphere.00114-25)
Supplement: Fig. S1 — Experimental pipeline. [file msphere.00114-25-s0001.pdf]

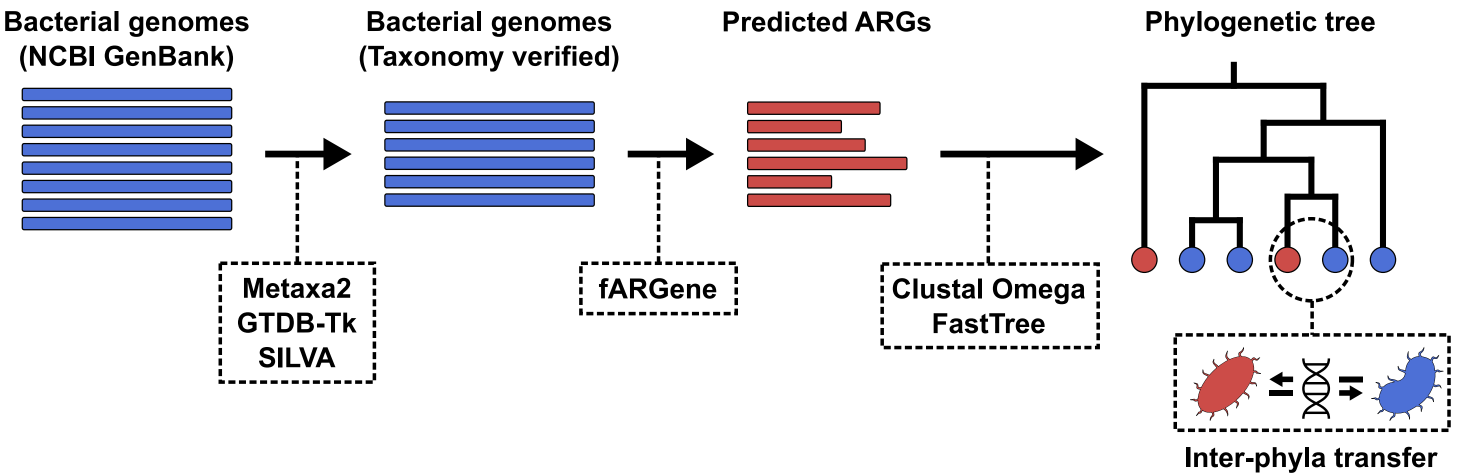
Bacterial genomes (NCBI GenBank)
Bacterial genomes (Taxonomy verified)
Predicted ARGs
Phylogenetic tree
Metaxa2
GTDB-Tk
SILVA
fARGene
Clustal Omega
FastTree
Inter-phyla transfer
