# Supplementary material for: The transfer of antibiotic resistance genes between evolutionarily distant bacteria
Source: mSphere. 2025 Jun 3;10(6):e00114-25. doi: 10.1128/msphere.00114-25 (PMC12188727; doi:10.1128/msphere.00114-25)
Supplement: Fig. S20 — Mobile element types and co-localized antibiotic resistance genes involved in inter-phylum transfers. [file msphere.00114-25-s0003.pdf]

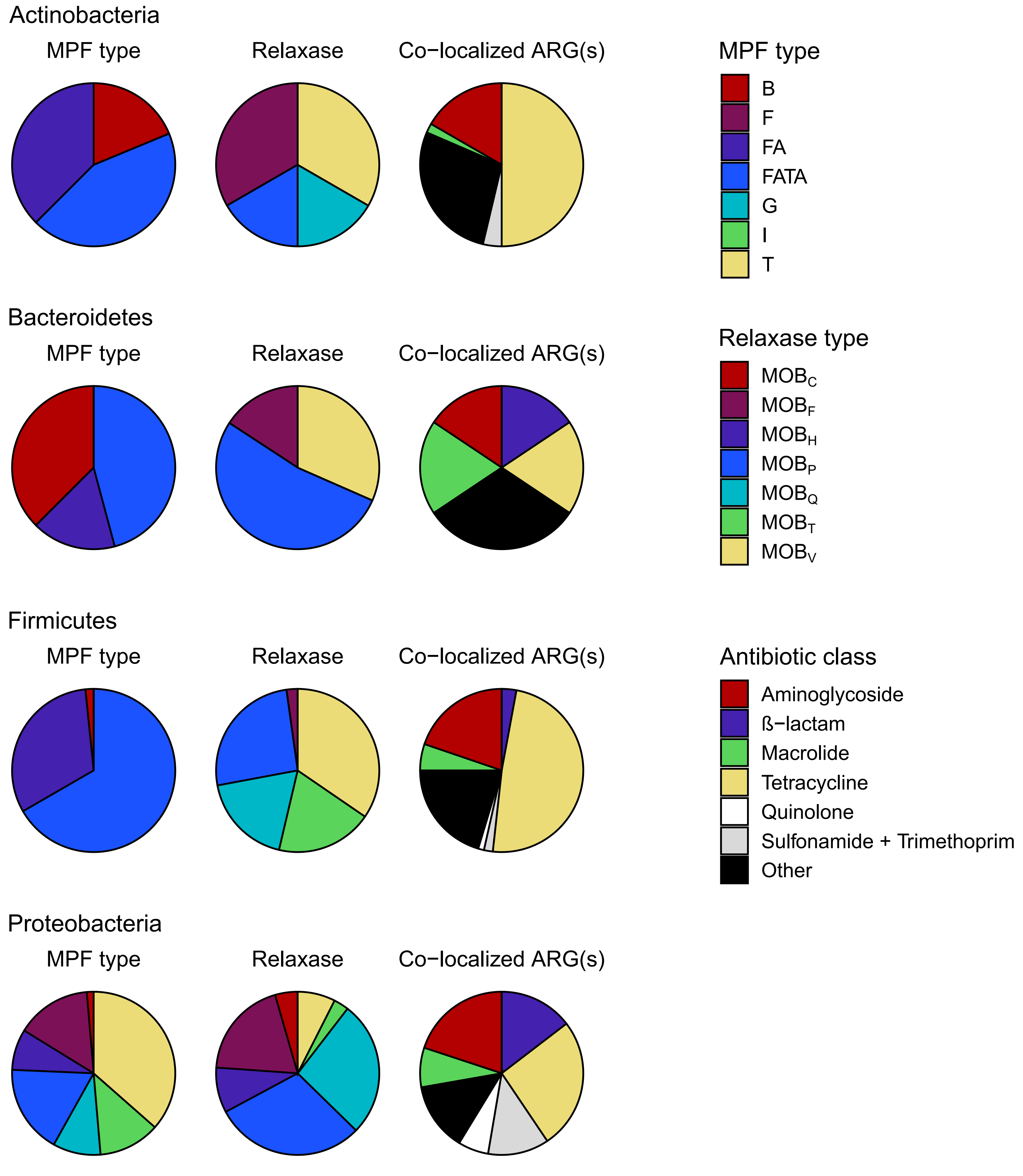
Actinobacteria
MPF type
Relaxase
Co−localized ARG(s)
Bacteroidetes
MPF type
Relaxase
Co−localized ARG(s)
Firmicutes
MPF type
Relaxase
Co−localized ARG(s)
Proteobacteria
MPF type
Relaxase
Co−localized ARG(s)
MPF type
B
F
FA
FATA
G
I
T
Relaxase type
MOB_C
MOB_F
MOB_H
MOB_P
MOB_Q
MOB_T
MOB_V
Antibiotic class
Aminoglycoside
ß−lactam
Macrolide
Tetracycline
Quinolone
Sulfonamide + Trimethoprim
Other
