# Supplementary material for: The transfer of antibiotic resistance genes between evolutionarily distant bacteria
Source: mSphere. 2025 Jun 3;10(6):e00114-25. doi: 10.1128/msphere.00114-25 (PMC12188727; doi:10.1128/msphere.00114-25)
Supplement: Legends — Supplemental material legends. [file msphere.00114-25-s0008.docx]

**S1 Table. Total number of removed sequences of taxonomy information for each class after following our curation criteria.** Sequences were kept in the alignment but did not compute for the evaluation of events.

**S2 Table.** Isolation sources scrutinized from the NCBI nucleotide database categorized into five major groups: soil, sediment, water, human, animal, and others.

**S3 Table.** Isolation source exclusion criteria.

**S1 Fig. Experimental pipeline.** Overview of the computational workflow used to identify potential horizontal gene transfer (HGT) events of antibiotic resistance genes (ARGs) across bacterial phyla. Bacterial genomes were first obtained from NCBI GenBank and taxonomically verified using Metaxa2, GTDB-Tk, and SILVA databases. ARGs were predicted from the verified genomes using fARGene. The predicted ARGs were then aligned and used to construct phylogenetic trees with Clustal Omega and FastTree. Finally, inter-phyla transfer events were inferred based on the phylogenetic clustering of ARGs from distantly related taxa.

**S2–19 Figs. Circular phylogenetic trees representing each class of antibiotic resistance genes (ARGs).** Maximum-likelihood phylogenetic trees were constructed for individual ARG families using predicted sequences from taxonomically verified bacterial genomes. Each tree is annotated with outer rings showing (1) the taxonomic classification of host organisms at the phylum level and (2) BLAST hit identity (%) against known ARG sequences. Red asterisks or symbols highlight putative inter-phyla horizontal gene transfer (HGT) events.

**S20 Fig. Mobile element types and co-localized antibiotic resistance genes (ARGs) involved in inter-phyla transfers.** Pie charts show the distribution of MPF types (left), relaxase types (middle), and ARG classes (right) found with ARGs involved in inter-phyla transfers (IPTs) in four major bacterial phyla: Actinobacteria, Bacteroidetes, Firmicutes, and Proteobacteria.

**S21–S38 Figs. Environmental distribution and transfer frequency of antibiotic resistance gene (ARG) classes.** Donut charts show the association of individual ARG families with different environments based on available genome metadata. The inner ring represents the proportion of genomes carrying a given ARG found in each environment (e.g., human, soil, water), while the outer ring shows the proportion of those genomes involved in inter-phyla transfer (IPT) events.
